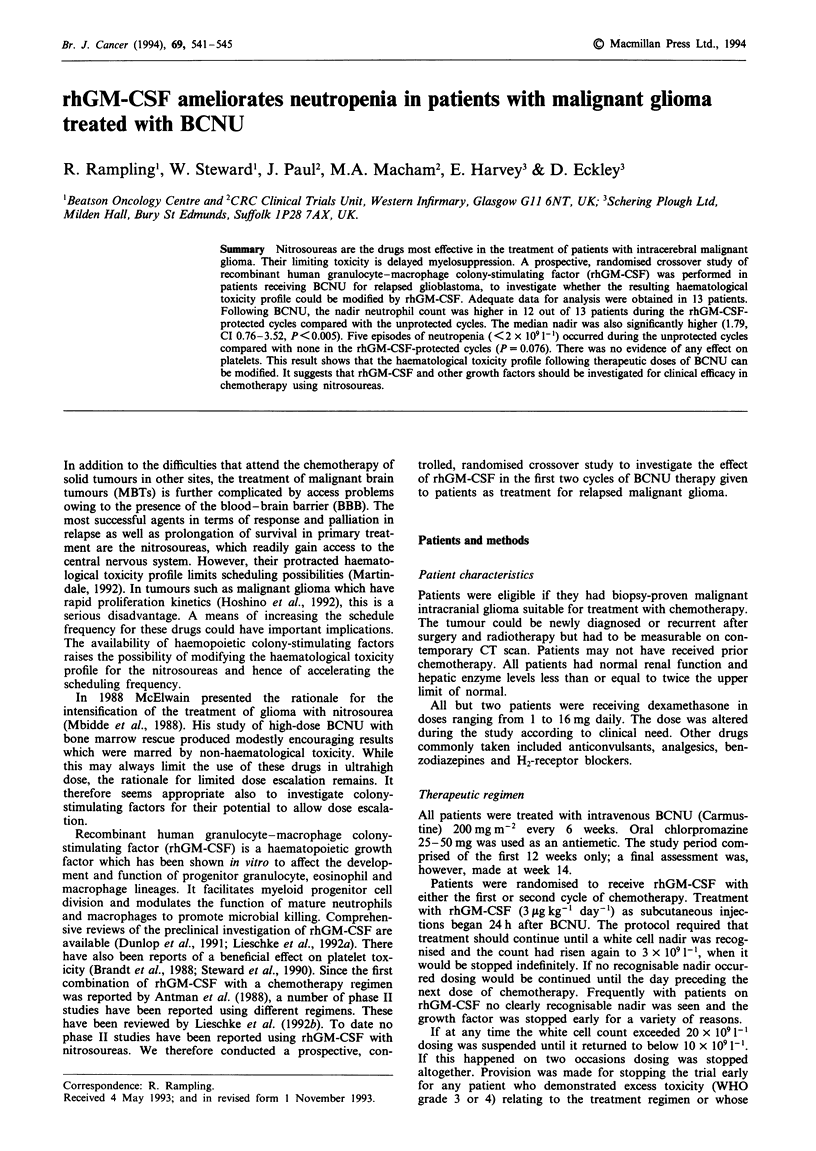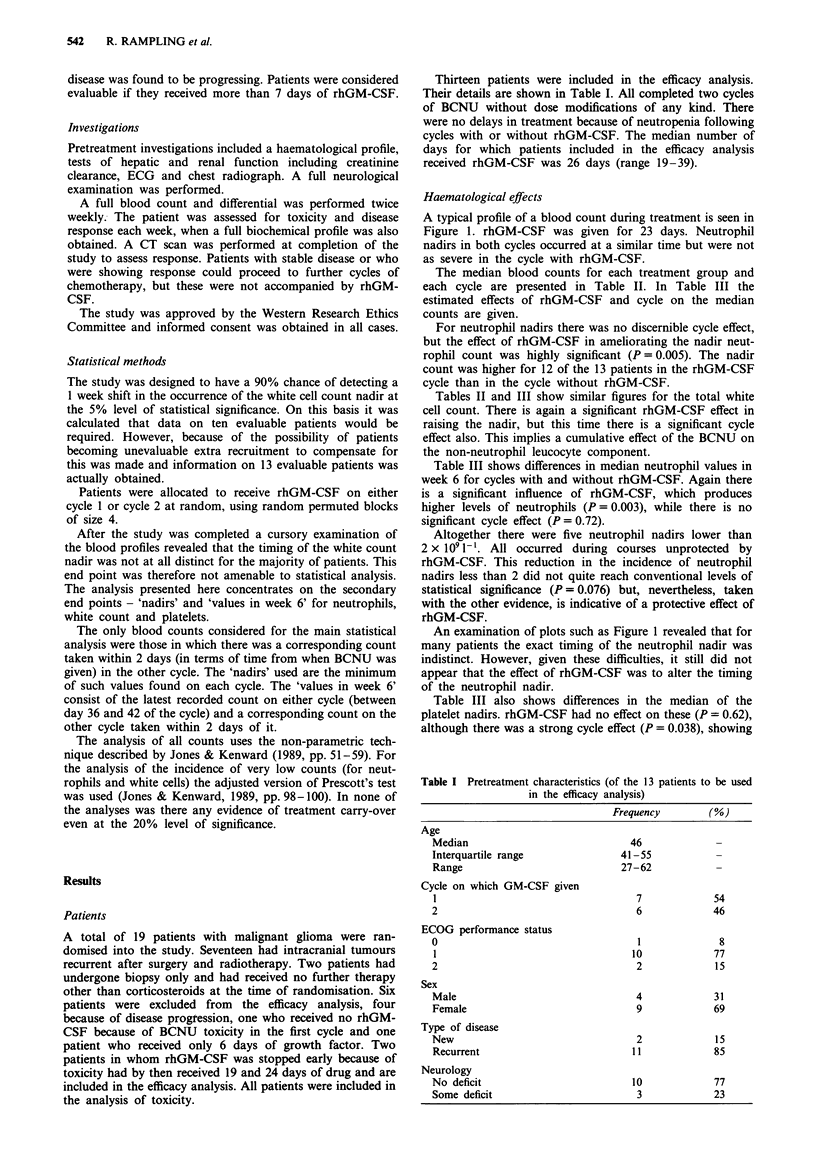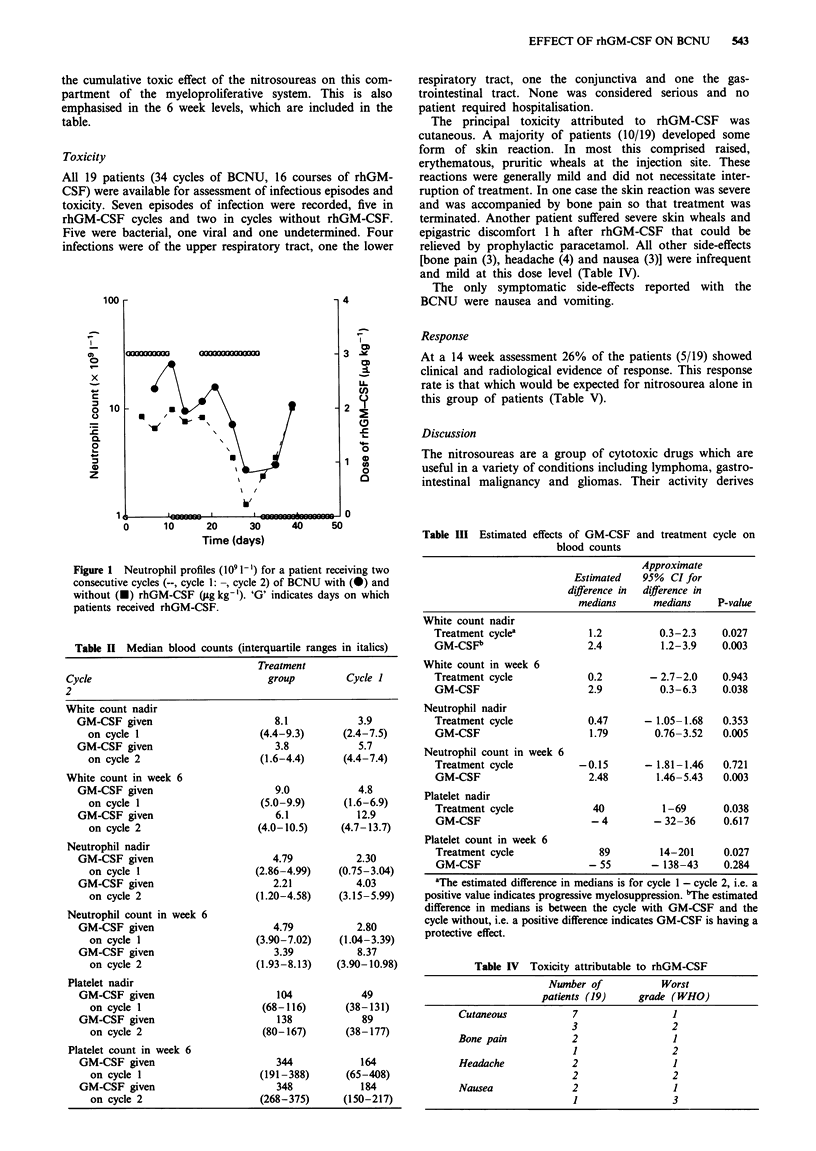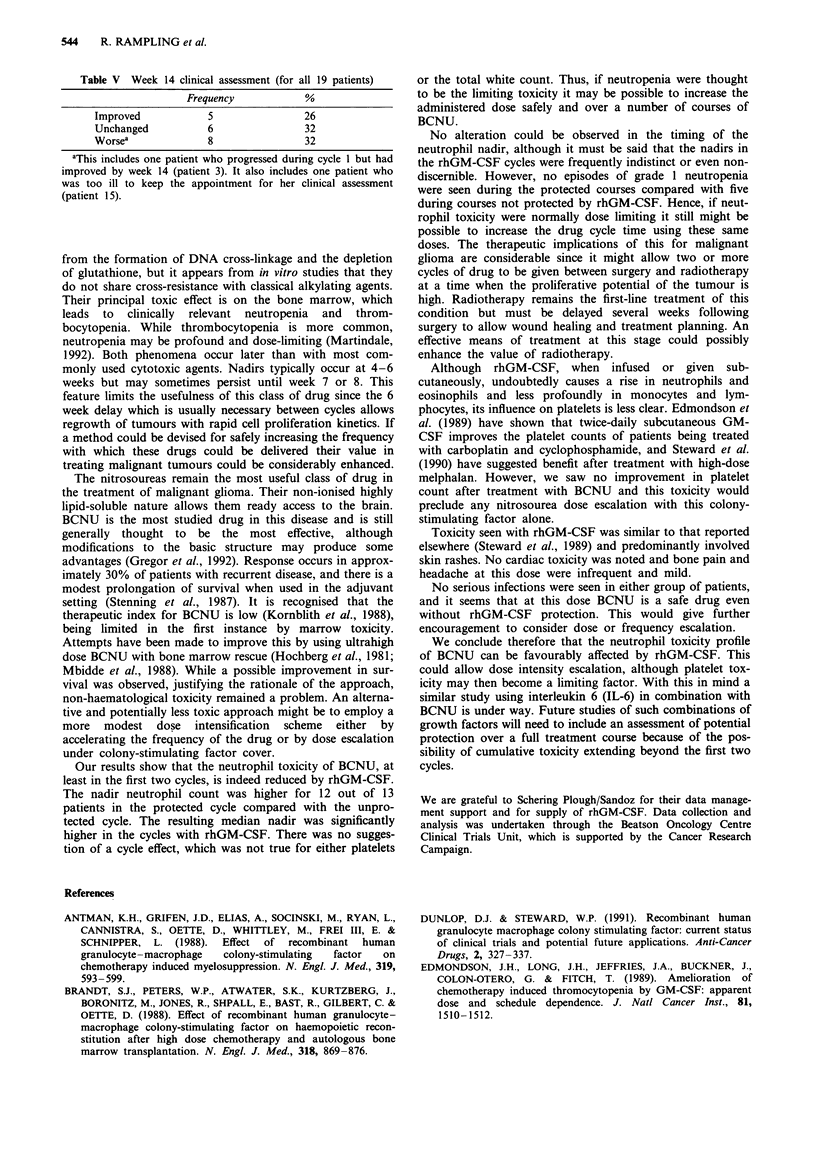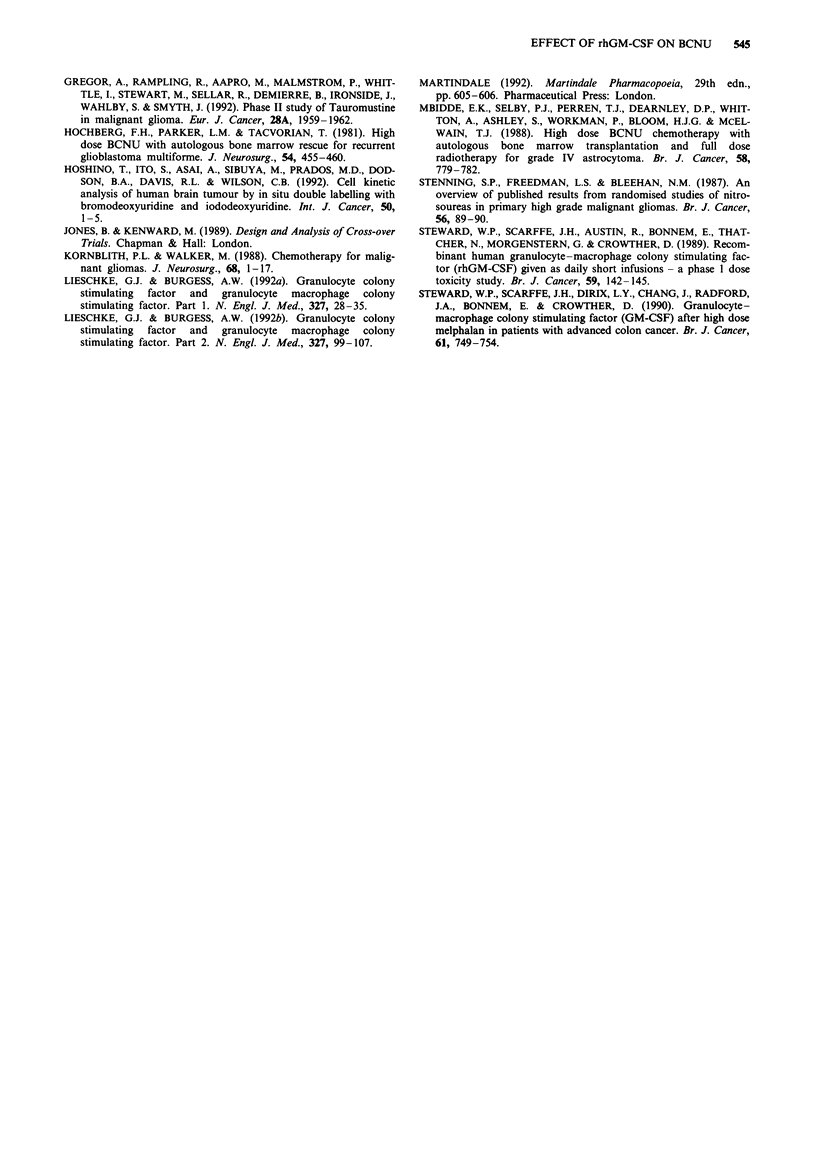# rhGM-CSF ameliorates neutropenia in patients with malignant glioma treated with BCNU.

**DOI:** 10.1038/bjc.1994.98

**Published:** 1994-03

**Authors:** R. Rampling, W. Steward, J. Paul, M. A. Macham, E. Harvey, D. Eckley

**Affiliations:** Beatson Oncology Centre, Western Infirmary, Glasgow, UK.

## Abstract

Nitrosoureas are the drugs most effective in the treatment of patients with intracerebral malignant glioma. Their limiting toxicity is delayed myelosuppression. A prospective, randomised crossover study of recombinant human granulocyte-macrophage colony-stimulating factor (rhGM-CSF) was performed in patients receiving BCNU for relapsed glioblastoma, to investigate whether the resulting haematological toxicity profile could be modified by rhGM-CSF. Adequate data for analysis were obtained in 13 patients. Following BCNU, the nadir neutrophil count was higher in 12 out of 13 patients during the rhGM-CSF-protected cycles compared with the unprotected cycles. The median nadir was also significantly higher (1.79, CI 0.76-3.52, P < 0.005). Five episodes of neutropenia (< 2 x 10(9) l-1) occurred during the unprotected cycles compared with none in the rhGM-CSF-protected cycles (P = 0.076). There was no evidence of any effect on platelets. This result shows that the haematological toxicity profile following therapeutic doses of BCNU can be modified. It suggests that rhGM-CSF and other growth factors should be investigated for clinical efficacy in chemotherapy using nitrosoureas.


Br. J. Cancer (1994), 69, 541-545 © Macmillan Press Ltd., 1994

### rhGM-CSF ameliorates neutropenia in patients with malignant glioma treated with BCNU

R. Rampling[1], W. Steward[1], J. Paul[2], M.A. Macham[2], E. Harvey[3] & D. Eckley[3]

[1]Beatson Oncology Centre and [2]CRC Clinical Trials Unit, Western Infirmary, Glasgow G11 6NT, UK; [3]Schering Plough Ltd, Milden Hall, Bury St Edmunds, Suffolk 1P28 7AX, UK.

**Summary** Nitrosoureas are the drugs most effective in the treatment of patients with intracerebral malignant glioma. Their limiting toxicity is delayed myelosuppression. A prospective, randomised crossover study of recombinant human granulocyte-macrophage colony-stimulating factor (rhGM-CSF) was performed in patients receiving BCNU for relapsed glioblastoma, to investigate whether the resulting haematological toxicity profile could be modified by rhGM-CSF. Adequate data for analysis were obtained in 13 patients. Following BCNU, the nadir neutrophil count was higher in 12 out of 13 patients during the rhGM-CSF-protected cycles compared with the unprotected cycles. The median nadir was also significantly higher (1.79, CI 0.76-3.52, $P < 0.005$). Five episodes of neutropenia ($< 2 \times 10^9 l^{-1}$) occurred during the unprotected cycles compared with none in the rhGM-CSF-protected cycles ($P = 0.076$). There was no evidence of any effect on platelets. This result shows that the haematological toxicity profile following therapeutic doses of BCNU can be modified. It suggests that rhGM-CSF and other growth factors should be investigated for clinical efficacy in chemotherapy using nitrosoureas.

In addition to the difficulties that attend the chemotherapy of solid tumours in other sites, the treatment of malignant brain tumours (MBTs) is further complicated by access problems owing to the presence of the blood-brain barrier (BBB). The most successful agents in terms of response and palliation in relapse as well as prolongation of survival in primary treatment are the nitrosoureas, which readily gain access to the central nervous system. However, their protracted haematological toxicity profile limits scheduling possibilities (Martindale, 1992). In tumours such as malignant glioma which have rapid proliferation kinetics (Hoshino *et al.*, 1992), this is a serious disadvantage. A means of increasing the schedule frequency for these drugs could have important implications. The availability of haemopoietic colony-stimulating factors raises the possibility of modifying the haematological toxicity profile for the nitrosoureas and hence of accelerating the scheduling frequency.

In 1988 McElwain presented the rationale for the intensification of the treatment of glioma with nitrosourea (Mbidde *et al.*, 1988). His study of high-dose BCNU with bone marrow rescue produced modestly encouraging results which were marred by non-haematological toxicity. While this may always limit the use of these drugs in ultrahigh dose, the rationale for limited dose escalation remains. It therefore seems appropriate also to investigate colony-stimulating factors for their potential to allow dose escalation.

Recombinant human granulocyte-macrophage colony-stimulating factor (rhGM-CSF) is a haematopoietic growth factor which has been shown *in vitro* to affect the development and function of progenitor granulocyte, eosinophil and macrophage lineages. It facilitates myeloid progenitor cell division and modulates the function of mature neutrophils and macrophages to promote microbial killing. Comprehensive reviews of the preclinical investigation of rhGM-CSF are available (Dunlop *et al.*, 1991; Lieschke *et al.*, 1992*a*). There have also been reports of a beneficial effect on platelet toxicity (Brandt *et al.*, 1988; Steward *et al.*, 1990). Since the first combination of rhGM-CSF with a chemotherapy regimen was reported by Antman *et al.* (1988), a number of phase II studies have been reported using different regimens. These have been reviewed by Lieschke *et al.* (1992*b*). To date no phase II studies have been reported using rhGM-CSF with nitrosoureas. We therefore conducted a prospective, controlled, randomised crossover study to investigate the effect of rhGM-CSF in the first two cycles of BCNU therapy given to patients as treatment for relapsed malignant glioma.

Correspondence: R. Rampling.
Received 4 May 1993; and in revised form 1 November 1993.

### Patients and methods

### Patient characteristics

Patients were eligible if they had biopsy-proven malignant intracranial glioma suitable for treatment with chemotherapy. The tumour could be newly diagnosed or recurrent after surgery and radiotherapy but had to be measurable on contemporary CT scan. Patients may not have received prior chemotherapy. All patients had normal renal function and hepatic enzyme levels less than or equal to twice the upper limit of normal.

All but two patients were receiving dexamethasone in doses ranging from 1 to 16 mg daily. The dose was altered during the study according to clinical need. Other drugs commonly taken included anticonvulsants, analgesics, benzodiazepines and $H_2$-receptor blockers.

### Therapeutic regimen

All patients were treated with intravenous BCNU (Carmustine) 200 mg $m^{-2}$ every 6 weeks. Oral chlorpromazine 25-50 mg was used as an antiemetic. The study period comprised of the first 12 weeks only; a final assessment was, however, made at week 14.

Patients were randomised to receive rhGM-CSF with either the first or second cycle of chemotherapy. Treatment with rhGM-CSF (3 $\mu g\ kg^{-1}\ day^{-1}$) as subcutaneous injections began 24 h after BCNU. The protocol required that treatment should continue until a white cell nadir was recognised and the count had risen again to $3 \times 10^9 l^{-1}$, when it would be stopped indefinitely. If no recognisable nadir occurred dosing would be continued until the day preceding the next dose of chemotherapy. Frequently with patients on rhGM-CSF no clearly recognisable nadir was seen and the growth factor was stopped early for a variety of reasons.

If at any time the white cell count exceeded $20 \times 10^9 l^{-1}$ dosing was suspended until it returned to below $10 \times 10^9 l^{-1}$. If this happened on two occasions dosing was stopped altogether. Provision was made for stopping the trial early for any patient who demonstrated excess toxicity (WHO grade 3 or 4) relating to the treatment regimen or whose disease was found to be progressing. Patients were considered evaluable if they received more than 7 days of rhGM-CSF.

*Investigations*

Pretreatment investigations included a haematological profile, tests of hepatic and renal function including creatinine clearance, ECG and chest radiograph. A full neurological examination was performed.

A full blood count and differential was performed twice weekly. The patient was assessed for toxicity and disease response each week, when a full biochemical profile was also obtained. A CT scan was performed at completion of the study to assess response. Patients with stable disease or who were showing response could proceed to further cycles of chemotherapy, but these were not accompanied by rhGM-CSF.

The study was approved by the Western Research Ethics Committee and informed consent was obtained in all cases.

*Statistical methods*

The study was designed to have a 90% chance of detecting a 1 week shift in the occurrence of the white cell count nadir at the 5% level of statistical significance. On this basis it was calculated that data on ten evaluable patients would be required. However, because of the possibility of patients becoming unevaluable extra recruitment to compensate for this was made and information on 13 evaluable patients was actually obtained.

Patients were allocated to receive rhGM-CSF on either cycle 1 or cycle 2 at random, using random permuted blocks of size 4.

After the study was completed a cursory examination of the blood profiles revealed that the timing of the white count nadir was not at all distinct for the majority of patients. This end point was therefore not amenable to statistical analysis. The analysis presented here concentrates on the secondary end points – 'nadirs' and 'values in week 6' for neutrophils, white count and platelets.

The only blood counts considered for the main statistical analysis were those in which there was a corresponding count taken within 2 days (in terms of time from when BCNU was given) in the other cycle. The 'nadirs' used are the minimum of such values found on each cycle. The 'values in week 6' consist of the latest recorded count on either cycle (between day 36 and 42 of the cycle) and a corresponding count on the other cycle taken within 2 days of it.

The analysis of all counts uses the non-parametric technique described by Jones & Kenward (1989, pp. 51–59). For the analysis of the incidence of very low counts (for neutrophils and white cells) the adjusted version of Prescott's test was used (Jones & Kenward, 1989, pp. 98–100). In none of the analyses was there any evidence of treatment carry-over even at the 20% level of significance.

### Results

*Patients*

A total of 19 patients with malignant glioma were randomised into the study. Seventeen had intracranial tumours recurrent after surgery and radiotherapy. Two patients had undergone biopsy only and had received no further therapy other than corticosteroids at the time of randomisation. Six patients were excluded from the efficacy analysis, four because of disease progression, one who received no rhGM-CSF because of BCNU toxicity in the first cycle and one patient who received only 6 days of growth factor. Two patients in whom rhGM-CSF was stopped early because of toxicity had by then received 19 and 24 days of drug and are included in the efficacy analysis. All patients were included in the analysis of toxicity.

Thirteen patients were included in the efficacy analysis. Their details are shown in Table I. All completed two cycles of BCNU without dose modifications of any kind. There were no delays in treatment because of neutropenia following cycles with or without rhGM-CSF. The median number of days for which patients included in the efficacy analysis received rhGM-CSF was 26 days (range 19–39).

*Haematological effects*

A typical profile of a blood count during treatment is seen in Figure 1. rhGM-CSF was given for 23 days. Neutrophil nadirs in both cycles occurred at a similar time but were not as severe in the cycle with rhGM-CSF.

The median blood counts for each treatment group and each cycle are presented in Table II. In Table III the estimated effects of rhGM-CSF and cycle on the median counts are given.

For neutrophil nadirs there was no discernible cycle effect, but the effect of rhGM-CSF in ameliorating the nadir neutrophil count was highly significant ($P = 0.005$). The nadir count was higher for 12 of the 13 patients in the rhGM-CSF cycle than in the cycle without rhGM-CSF.

Tables II and III show similar figures for the total white cell count. There is again a significant rhGM-CSF effect in raising the nadir, but this time there is a significant cycle effect also. This implies a cumulative effect of the BCNU on the non-neutrophil leucocyte component.

Table III shows differences in median neutrophil values in week 6 for cycles with and without rhGM-CSF. Again there is a significant influence of rhGM-CSF, which produces higher levels of neutrophils ($P = 0.003$), while there is no significant cycle effect ($P = 0.72$).

Altogether there were five neutrophil nadirs lower than $2 \times 10^9 l^{-1}$. All occurred during courses unprotected by rhGM-CSF. This reduction in the incidence of neutrophil nadirs less than 2 did not quite reach conventional levels of statistical significance ($P = 0.076$) but, nevertheless, taken with the other evidence, is indicative of a protective effect of rhGM-CSF.

An examination of plots such as Figure 1 revealed that for many patients the exact timing of the neutrophil nadir was indistinct. However, given these difficulties, it still did not appear that the effect of rhGM-CSF was to alter the timing of the neutrophil nadir.

Table III also shows differences in the median of the platelet nadirs. rhGM-CSF had no effect on these ($P = 0.62$), although there was a strong cycle effect ($P = 0.038$), showing

**Table I** Pretreatment characteristics (of the 13 patients to be used in the efficacy analysis)

| | *Frequency* | *(%)* |
|---|---|---|
| Age | | |
| Median | 46 | – |
| Interquartile range | 41–55 | – |
| Range | 27–62 | – |
| Cycle on which GM-CSF given | | |
| 1 | 7 | 54 |
| 2 | 6 | 46 |
| ECOG performance status | | |
| 0 | 1 | 8 |
| 1 | 10 | 77 |
| 2 | 2 | 15 |
| Sex | | |
| Male | 4 | 31 |
| Female | 9 | 69 |
| Type of disease | | |
| New | 2 | 15 |
| Recurrent | 11 | 85 |
| Neurology | | |
| No deficit | 10 | 77 |
| Some deficit | 3 | 23 |

the cumulative toxic effect of the nitrosoureas on this compartment of the myeloproliferative system. This is also emphasised in the 6 week levels, which are included in the table.

### *Toxicity*

All 19 patients (34 cycles of BCNU, 16 courses of rhGM-CSF) were available for assessment of infectious episodes and toxicity. Seven episodes of infection were recorded, five in rhGM-CSF cycles and two in cycles without rhGM-CSF. Five were bacterial, one viral and one undetermined. Four infections were of the upper respiratory tract, one the lower respiratory tract, one the conjunctiva and one the gastrointestinal tract. None was considered serious and no patient required hospitalisation.

The principal toxicity attributed to rhGM-CSF was cutaneous. A majority of patients (10/19) developed some form of skin reaction. In most this comprised raised, erythematous, pruritic wheals at the injection site. These reactions were generally mild and did not necessitate interruption of treatment. In one case the skin reaction was severe and was accompanied by bone pain so that treatment was terminated. Another patient suffered severe skin wheals and epigastric discomfort 1 h after rhGM-CSF that could be relieved by prophylactic paracetamol. All other side-effects [bone pain (3), headache (4) and nausea (3)] were infrequent and mild at this dose level (Table IV).

The only symptomatic side-effects reported with the BCNU were nausea and vomiting.

**Figure 1** Neutrophil profiles ($10^9\ l^{-1}$) for a patient receiving two consecutive cycles (--, cycle 1: –, cycle 2) of BCNU with (●) and without (■) rhGM-CSF ($\mu g\ kg^{-1}$). 'G' indicates days on which patients received rhGM-CSF.

### *Response*

At a 14 week assessment 26% of the patients (5/19) showed clinical and radiological evidence of response. This response rate is that which would be expected for nitrosourea alone in this group of patients (Table V).

### *Discussion*

The nitrosoureas are a group of cytotoxic drugs which are useful in a variety of conditions including lymphoma, gastrointestinal malignancy and gliomas. Their activity derives

**Table II** Median blood counts (interquartile ranges in italics)

| *Cycle 2* | *Treatment group* | *Cycle 1* |
|---|---|---|
| White count nadir | | |
| GM-CSF given | 8.1 | 3.9 |
| on cycle 1 | (4.4–9.3) | (2.4–7.5) |
| GM-CSF given | 3.8 | 5.7 |
| on cycle 2 | (1.6–4.4) | (4.4–7.4) |
| White count in week 6 | | |
| GM-CSF given | 9.0 | 4.8 |
| on cycle 1 | (5.0–9.9) | (1.6–6.9) |
| GM-CSF given | 6.1 | 12.9 |
| on cycle 2 | (4.0–10.5) | (4.7–13.7) |
| Neutrophil nadir | | |
| GM-CSF given | 4.79 | 2.30 |
| on cycle 1 | (2.86–4.99) | (0.75–3.04) |
| GM-CSF given | 2.21 | 4.03 |
| on cycle 2 | (1.20–4.58) | (3.15–5.99) |
| Neutrophil count in week 6 | | |
| GM-CSF given | 4.79 | 2.80 |
| on cycle 1 | (3.90–7.02) | (1.04–3.39) |
| GM-CSF given | 3.39 | 8.37 |
| on cycle 2 | (1.93–8.13) | (3.90–10.98) |
| Platelet nadir | | |
| GM-CSF given | 104 | 49 |
| on cycle 1 | (68–116) | (38–131) |
| GM-CSF given | 138 | 89 |
| on cycle 2 | (80–167) | (38–177) |
| Platelet count in week 6 | | |
| GM-CSF given | 344 | 164 |
| on cycle 1 | (191–388) | (65–408) |
| GM-CSF given | 348 | 184 |
| on cycle 2 | (268–375) | (150–217) |

**Table III** Estimated effects of GM-CSF and treatment cycle on blood counts

| | *Estimated difference in medians* | *Approximate 95% CI for difference in medians* | *P-value* |
|---|---|---|---|
| White count nadir | | | |
| Treatment cycle[a] | 1.2 | 0.3–2.3 | 0.027 |
| GM-CSF[b] | 2.4 | 1.2–3.9 | 0.003 |
| White count in week 6 | | | |
| Treatment cycle | 0.2 | – 2.7–2.0 | 0.943 |
| GM-CSF | 2.9 | 0.3–6.3 | 0.038 |
| Neutrophil nadir | | | |
| Treatment cycle | 0.47 | – 1.05–1.68 | 0.353 |
| GM-CSF | 1.79 | 0.76–3.52 | 0.005 |
| Neutrophil count in week 6 | | | |
| Treatment cycle | –0.15 | – 1.81–1.46 | 0.721 |
| GM-CSF | 2.48 | 1.46–5.43 | 0.003 |
| Platelet nadir | | | |
| Treatment cycle | 40 | 1–69 | 0.038 |
| GM-CSF | – 4 | – 32–36 | 0.617 |
| Platelet count in week 6 | | | |
| Treatment cycle | 89 | 14–201 | 0.027 |
| GM-CSF | – 55 | – 138–43 | 0.284 |

[a]The estimated difference in medians is for cycle 1 – cycle 2, i.e. a positive value indicates progressive myelosuppression. [b]The estimated difference in medians is between the cycle with GM-CSF and the cycle without, i.e. a positive difference indicates GM-CSF is having a protective effect.

**Table IV** Toxicity attributable to rhGM-CSF

| | *Number of patients (19)* | *Worst grade (WHO)* |
|---|---|---|
| *Cutaneous* | *7* | *1* |
| | *3* | *2* |
| *Bone pain* | *2* | *1* |
| | *1* | *2* |
| *Headache* | *2* | *1* |
| | *2* | *2* |
| *Nausea* | *2* | *1* |
| | *1* | *3* |

Table V Week 14 clinical assessment (for all 19 patients)

| | Frequency | % |
|---|---|---|
| Improved | 5 | 26 |
| Unchanged | 6 | 32 |
| Worse[a] | 8 | 32 |

[a]This includes one patient who progressed during cycle 1 but had improved by week 14 (patient 3). It also includes one patient who was too ill to keep the appointment for her clinical assessment (patient 15).

from the formation of DNA cross-linkage and the depletion of glutathione, but it appears from *in vitro* studies that they do not share cross-resistance with classical alkylating agents. Their principal toxic effect is on the bone marrow, which leads to clinically relevant neutropenia and thrombocytopenia. While thrombocytopenia is more common, neutropenia may be profound and dose-limiting (Martindale, 1992). Both phenomena occur later than with most commonly used cytotoxic agents. Nadirs typically occur at 4-6 weeks but may sometimes persist until week 7 or 8. This feature limits the usefulness of this class of drug since the 6 week delay which is usually necessary between cycles allows regrowth of tumours with rapid cell proliferation kinetics. If a method could be devised for safely increasing the frequency with which these drugs could be delivered their value in treating malignant tumours could be considerably enhanced.

The nitrosoureas remain the most useful class of drug in the treatment of malignant glioma. Their non-ionised highly lipid-soluble nature allows them ready access to the brain. BCNU is the most studied drug in this disease and is still generally thought to be the most effective, although modifications to the basic structure may produce some advantages (Gregor *et al.*, 1992). Response occurs in approximately 30% of patients with recurrent disease, and there is a modest prolongation of survival when used in the adjuvant setting (Stenning *et al.*, 1987). It is recognised that the therapeutic index for BCNU is low (Kornblith *et al.*, 1988), being limited in the first instance by marrow toxicity. Attempts have been made to improve this by using ultrahigh dose BCNU with bone marrow rescue (Hochberg *et al.*, 1981; Mbidde *et al.*, 1988). While a possible improvement in survival was observed, justifying the rationale of the approach, non-haematological toxicity remained a problem. An alternative and potentially less toxic approach might be to employ a more modest dose intensification scheme either by accelerating the frequency of the drug or by dose escalation under colony-stimulating factor cover.

Our results show that the neutrophil toxicity of BCNU, at least in the first two cycles, is indeed reduced by rhGM-CSF. The nadir neutrophil count was higher for 12 out of 13 patients in the protected cycle compared with the unprotected cycle. The resulting median nadir was significantly higher in the cycles with rhGM-CSF. There was no suggestion of a cycle effect, which was not true for either platelets or the total white count. Thus, if neutropenia were thought to be the limiting toxicity it may be possible to increase the administered dose safely and over a number of courses of BCNU.

No alteration could be observed in the timing of the neutrophil nadir, although it must be said that the nadirs in the rhGM-CSF cycles were frequently indistinct or even non-discernible. However, no episodes of grade 1 neutropenia were seen during the protected courses compared with five during courses not protected by rhGM-CSF. Hence, if neutrophil toxicity were normally dose limiting it still might be possible to increase the drug cycle time using these same doses. The therapeutic implications of this for malignant glioma are considerable since it might allow two or more cycles of drug to be given between surgery and radiotherapy at a time when the proliferative potential of the tumour is high. Radiotherapy remains the first-line treatment of this condition but must be delayed several weeks following surgery to allow wound healing and treatment planning. An effective means of treatment at this stage could possibly enhance the value of radiotherapy.

Although rhGM-CSF, when infused or given subcutaneously, undoubtedly causes a rise in neutrophils and eosinophils and less profoundly in monocytes and lymphocytes, its influence on platelets is less clear. Edmondson *et al.* (1989) have shown that twice-daily subcutaneous GM-CSF improves the platelet counts of patients being treated with carboplatin and cyclophosphamide, and Steward *et al.* (1990) have suggested benefit after treatment with high-dose melphalan. However, we saw no improvement in platelet count after treatment with BCNU and this toxicity would preclude any nitrosourea dose escalation with this colony-stimulating factor alone.

Toxicity seen with rhGM-CSF was similar to that reported elsewhere (Steward *et al.*, 1989) and predominantly involved skin rashes. No cardiac toxicity was noted and bone pain and headache at this dose were infrequent and mild.

No serious infections were seen in either group of patients, and it seems that at this dose BCNU is a safe drug even without rhGM-CSF protection. This would give further encouragement to consider dose or frequency escalation.

We conclude therefore that the neutrophil toxicity profile of BCNU can be favourably affected by rhGM-CSF. This could allow dose intensity escalation, although platelet toxicity may then become a limiting factor. With this in mind a similar study using interleukin 6 (IL-6) in combination with BCNU is under way. Future studies of such combinations of growth factors will need to include an assessment of potential protection over a full treatment course because of the possibility of cumulative toxicity extending beyond the first two cycles.

We are grateful to Schering Plough/Sandoz for their data management support and for supply of rhGM-CSF. Data collection and analysis was undertaken through the Beatson Oncology Centre Clinical Trials Unit, which is supported by the Cancer Research Campaign.

### References

ANTMAN, K.H., GRIFEN, J.D., ELIAS, A., SOCINSKI, M., RYAN, L., CANNISTRA, S., OETTE, D., WHITTLEY, M., FREI III, E. & SCHNIPPER, L. (1988). Effect of recombinant human granulocyte-macrophage colony-stimulating factor on chemotherapy induced myelosuppression. *N. Engl. J. Med.*, **319**, 593-599.

BRANDT, S.J., PETERS, W.P., ATWATER, S.K., KURTZBERG, J., BORONITZ, M., JONES, R., SHPALL, E., BAST, R., GILBERT, C. & OETTE, D. (1988). Effect of recombinant human granulocyte-macrophage colony-stimulating factor on haemopoietic reconstitution after high dose chemotherapy and autologous bone marrow transplantation. *N. Engl. J. Med.*, **318**, 869-876.

DUNLOP, D.J. & STEWARD, W.P. (1991). Recombinant human granulocyte macrophage colony stimulating factor: current status of clinical trials and potential future applications. *Anti-Cancer Drugs*, **2**, 327-337.

EDMONDSON, J.H., LONG, J.H., JEFFRIES, J.A., BUCKNER, J., COLON-OTERO, G. & FITCH, T. (1989). Amelioration of chemotherapy induced thromocytopenia by GM-CSF: apparent dose and schedule dependence. *J. Natl Cancer Inst.*, **81**, 1510-1512.

GREGOR, A., RAMPLING, R., AAPRO, M., MALMSTROM, P., WHITTLE, I., STEWART, M., SELLAR, R., DEMIERRE, B., IRONSIDE, J., WAHLBY, S. & SMYTH, J. (1992). Phase II study of Tauromustine in malignant glioma. *Eur. J. Cancer*, **28A**, 1959–1962.

HOCHBERG, F.H., PARKER, L.M. & TACVORIAN, T. (1981). High dose BCNU with autologous bone marrow rescue for recurrent glioblastoma multiforme. *J. Neurosurg.*, **54**, 455–460.

HOSHINO, T., ITO, S., ASAI, A., SIBUYA, M., PRADOS, M.D., DODSON, B.A., DAVIS, R.L. & WILSON, C.B. (1992). Cell kinetic analysis of human brain tumour by in situ double labelling with bromodeoxyuridine and iododeoxyuridine. *Int. J. Cancer*, **50**, 1–5.

JONES, B. & KENWARD, M. (1989). *Design and Analysis of Cross-over Trials*. Chapman & Hall: London.

KORNBLITH, P.L. & WALKER, M. (1988). Chemotherapy for malignant gliomas. *J. Neurosurg.*, **68**, 1–17.

LIESCHKE, G.J. & BURGESS, A.W. (1992*a*). Granulocyte colony stimulating factor and granulocyte macrophage colony stimulating factor. Part 1. *N. Engl. J. Med.*, **327**, 28–35.

LIESCHKE, G.J. & BURGESS, A.W. (1992*b*). Granulocyte colony stimulating factor and granulocyte macrophage colony stimulating factor. Part 2. *N. Engl. J. Med.*, **327**, 99–107.

MARTINDALE (1992). *Martindale Pharmacopoeia*, 29th edn., pp. 605–606. Pharmaceutical Press: London.

MBIDDE, E.K., SELBY, P.J., PERREN, T.J., DEARNLEY, D.P., WHITTON, A., ASHLEY, S., WORKMAN, P., BLOOM, H.J.G. & MCELWAIN, T.J. (1988). High dose BCNU chemotherapy with autologous bone marrow transplantation and full dose radiotherapy for grade IV astrocytoma. *Br. J. Cancer*, **58**, 779–782.

STENNING, S.P., FREEDMAN, L.S. & BLEEHAN, N.M. (1987). An overview of published results from randomised studies of nitrosoureas in primary high grade malignant gliomas. *Br. J. Cancer*, **56**, 89–90.

STEWARD, W.P., SCARFFE, J.H., AUSTIN, R., BONNEM, E., THATCHER, N., MORGENSTERN, G. & CROWTHER, D. (1989). Recombinant human granulocyte–macrophage colony stimulating factor (rhGM-CSF) given as daily short infusions – a phase 1 dose toxicity study. *Br. J. Cancer*, **59**, 142–145.

STEWARD, W.P., SCARFFE, J.H., DIRIX, L.Y., CHANG, J., RADFORD, J.A., BONNEM, E. & CROWTHER, D. (1990). Granulocyte–macrophage colony stimulating factor (GM-CSF) after high dose melphalan in patients with advanced colon cancer. *Br. J. Cancer*, **61**, 749–754.